# A rare case of IgG4-related disease masquerading as periurethral malignancy and review of the literature

**DOI:** 10.3389/fimmu.2022.1077609

**Published:** 2023-01-30

**Authors:** Zhiru Zeng, Shasha Gao, Xiaoyong Lu

**Affiliations:** ^1^ Department of Rheumatology, the Second Affiliated Hospital of Zhejiang University School of Medicine, Hangzhou, Zhejiang, China; ^2^ Department of Endocrinology and Rheumatology, Anji Traditional Chinese Medical Hospital, Huzhou, Zhejiang, China

**Keywords:** immunoglobulin G4-related disease, urethra, periurethral mass, dysuria, contrast-enhanced ultrasound (CEUS)

## Abstract

Immunoglobulin G4-related disease (IgG4-RD) is an immune-mediated fibroinflammatory disease that typically manifests as mass lesions affecting almost any organ including the pancreas, lacrimal and salivary glands, liver, lung and kidney. However, IgG4-RD with urethra involvement is scarce. We describe a rare case of IgG4-RD involving the urethra mimicking urethral carcinoma and review the published literature. A 64-years-old female presented with progressive dysuria for more than 2 months. Pelvic gadolinium-enhanced magnetic resonance imaging revealed a huge mass encasing the urethra which showed obvious enhancement in the arterial phase. And contrast-enhanced ultrasound showed that the entire mass was heterogeneously enhanced and displayed a fast-forward and fast-out pattern, which was highly suggestive of malignant tumor. The diagnosis of IgG4-RD was finally established by ultrasound-guided transvaginal mass needle biopsy. The patient was treated with methylprednisolone and cyclophosphamide and dysuria disappeared in the first week of therapy. She has been followed up in our clinic for 1 year without recurrence. The diagnosis of IgG4-RD should be considered in the differential diagnosis of a periurethral mass. Ultrasound-guided transvaginal mass needle biopsy is a safe and well-established tissue sampling method and should be performed in order to avoid unnecessary surgery.

## Introduction

Immunoglobulin G4-related disease (IgG4-RD) is a chronic, systemic, immune-mediated fibroinflammatory disease that is characterized by swelling or mass formation in various organs with abundant tissue infiltration of IgG4-positive plasma cells. It was first described in the pancreas in 1961 by Sarles H et al. in a patient with hypergammaglobulinemia and chronic pancreatitis (1); however, it was not identified as a unified systemic disorder until 2003, when Kamisawa T et al. proposed the close relationship between autoimmune pancreatitis and extrapancreatic manifestations (2). Since then, the term IgG4-RD has been used and reported in virtually every organ including lacrimal glands, salivary glands, thyroid gland, retroperitoneum, lungs, kidneys, aorta and skin (3).

IgG4-RD involvement of the urinary tract is relatively uncommon and the majority of the reported cases were located in the kidney, ureter and prostate (4). Clinical manifestations include tubulointerstitial nephritis, membranous glomerulonephropathy, ureteral obstruction due to ureteral pseudotumor or retroperitoneal fibrosis and prostatitis. However, IgG4-RD occurring in the urethra is extremely rare and only a few cases of urethral IgG4-RD have been reported. Herein, we report a case of a 64-year-old female diagnosed with IgG4-RD in the urethra mimicking urethral carcinoma and made a comprehensive literature review.

## Case presentation

**Figure 1:**
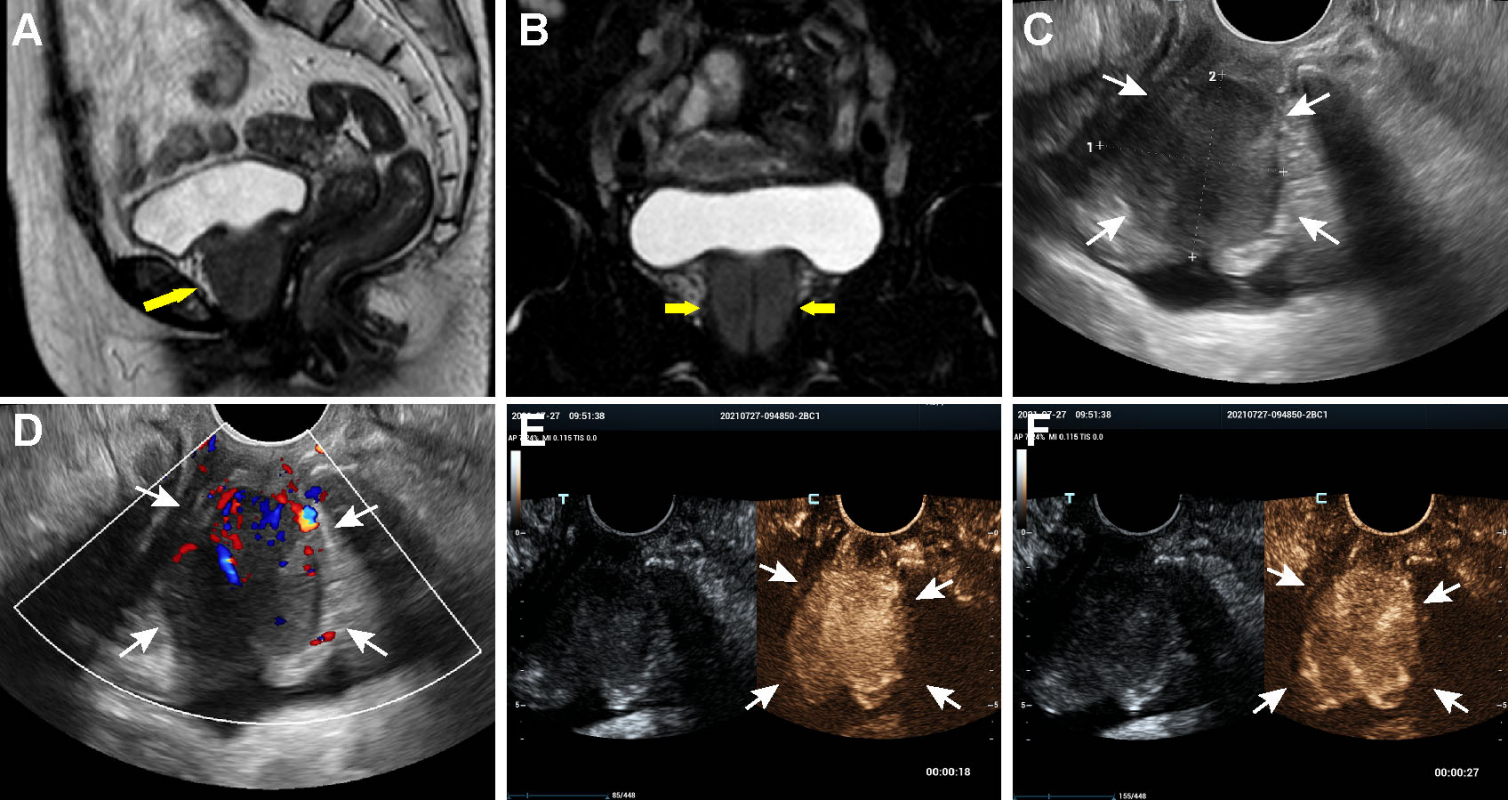
MRI, US and CEUS findings of the periurethral mass on admission. **(A, B)** Coronal and sagittal MRI of the pelvis, showing a well-defined mass around the urethra (arrows). **(C, D)** Transvaginal ultrasonography scan revealed a heterogeneously low echoic mass encasing urethra and color Doppler imaging indicated abundant blood flow signals in the lesion. **(E)** CEUS image obtained at the 18 sec after contrast injection indicating an overall heterogeneously high enhancement of the mass in the arterial phase. **(F)** CEUS image obtained at the 27 sec showed quick wash-out of the contrast in the lesion.

A 64-year-old female with a past medical history of asthma presented with progressive dysuria for 2 months. Pelvic gadolinium-enhanced magnetic resonance imaging (MRI) done at the local institution revealed 3.5×3.3×3.7 cm sized periurethral mass which extended from the distal urethra to the bladder neck (**Figures 1A, B**). And the mass showed low signal intensity at T1-weighted MRI, intermediate signal intensity at T2-weighted MRI and obvious enhancement in the arterial phase. In addition, multiple enlarged iliac lymph nodes were detected on both sides. Malignant tumor was suspected and the patient visited a urologist in our hospital for further diagnostic workup and management.

At admission, the patient was afebrile with mild, painless bilateral lacrimal and submandibular glands swelling. Physical examination revealed a fixed, hard mass bulging from the anterior vaginal wall, which was palpated through the vagina. Laboratory tests showed that peripheral eosinophil cell count was 0.15×10^9^/L, which was within the normal limits. Her serum IgE levels were high and reached 526.0 IU/mL (cut-off <100.0 IU/mL) while serum complement component C3 and C4 were both normal, measuring 0.88 g/L and 0.11 g/L respectively. Erythrocyte sedimentation rate (ESR) was elevated to 64 mm/h and C-reactive protein level was within the normal range. Her serum rheumatoid factor level was elevated to 142 IU/mL (cut-off <15.9 IU/mL) and antinuclear antibody (ANA) was detected at a titration of 1:160 but extractable nuclear antigens (ENAs), including anti-ribonucleoprotein, anti-smith antibody, anti-double stranded DNA, anti-Ro, and anti-La antibodies were all negative. Other tests (complete blood cell counts, creatinine, liver function test, thyroid function test, immunological test for HIV, HBsAg and HCV) were all normal and urine analysis results were not remarkable.

**Figure 2:**
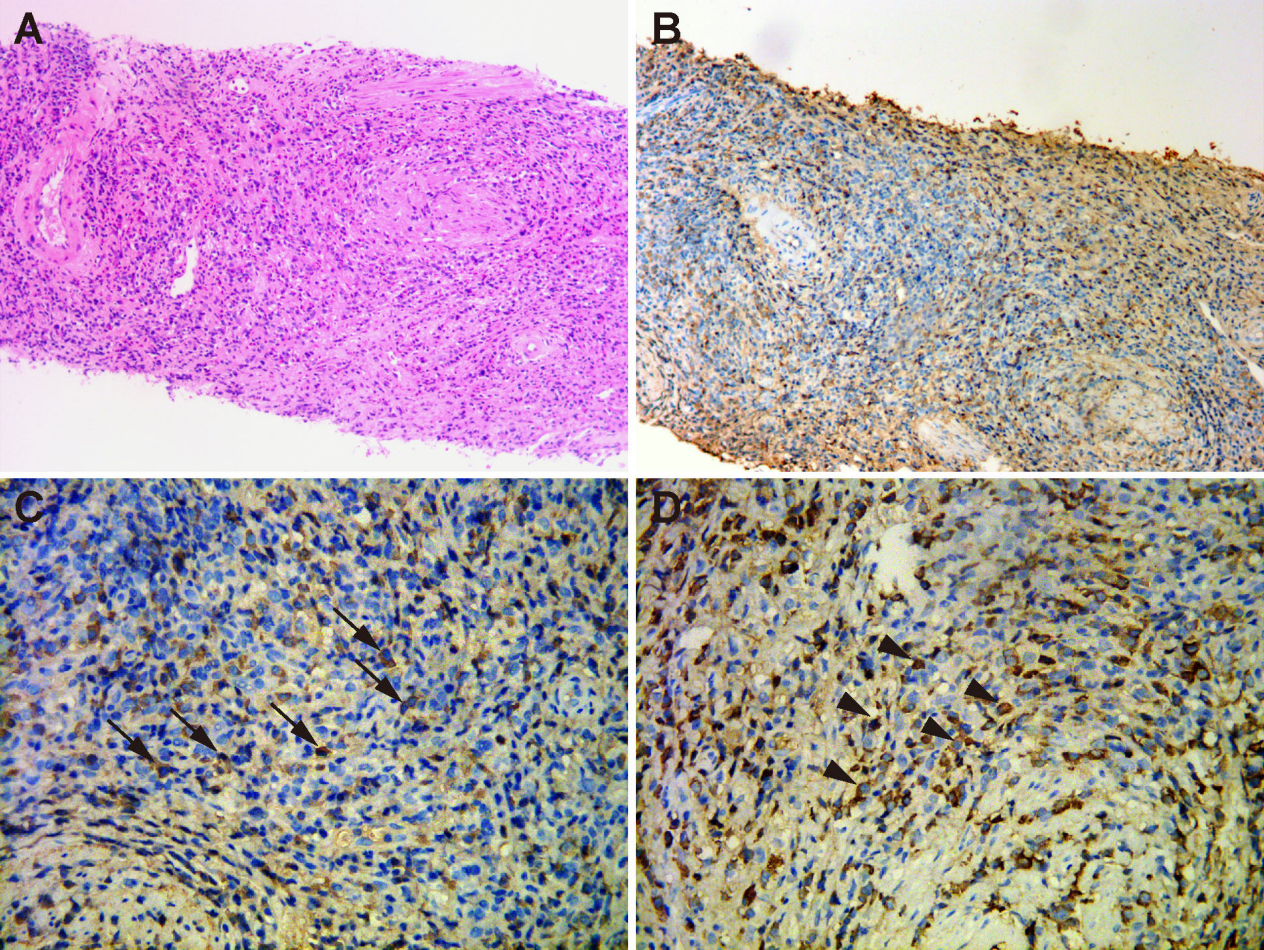
Histopathological examination of the periurethral mass. **(A)** Hematoxylin and eosin staining (×200) showed fibrous tissue with abundant infiltration of lymphocytes, plasma cells and eosinophils. **(B)** Immunohistochemical staining (×200) of IgG4-positive plasma cells. **(C)** Immunohistochemical staining (×400) showed that IgG4-positive plasma cells were above 50 per high power field (arrows). **(D)** Immunohistochemical staining (×400) revealed the infiltration of IgG-positive cells (arrow heads). IgG4/IgG ratio was 55.6%.

Then, the patient underwent transvaginal ultrasonography which revealed a large well-defined heterogeneous hypoechoic mass in front of the anterior vaginal wall, measuring 3.7×3.1×3.5 cm (**Figure 1C**). Color Doppler examination demonstrated abundant blood flow signals inside the mass (**Figure 1D**). To further characterize the lesion, contrast-enhanced ultrasound (CEUS) was performed. The contrast agent, SonoVue (Sulphur hexafluoride microbubbles) was injected intravenously and a timer was started following injection. Dual-image mode with grayscale and contrast images was obtained for optimal visualization of the lesion. It turned out that the contrast agent began to wash into the mass in 15 seconds after injection and the mass appeared overall heterogeneously high enhanced and displayed a fast-forward and fast-out pattern (**Figures 1E, F**). Based on these observations on CEUS, a radiologist with over 10-year experience pointed out that the mass tended to be malignant. Subsequently, the solid mass with malignancy suspicion received transvaginal ultrasound-guided core needle biopsy. Surprisingly, histological examination revealed no evidence of malignancy but fibrous tissue with diffuse infiltration of lymphocytes, plasma cells and eosinophils (**Figure 2A**). Further immunohistochemistry staining revealed that IgG4-positive plasma cells count was over 50/high power field (HPF) and the IgG4+/IgG ratio was approximately 55.6% (**Figures 2B–D**). In addition, the tissue was stained positive for Ki-67 (30%+), CD34 and CD31 but negative for cytokeratin (CK), anaplastic lymphoma kinase (ALK), smooth muscle actin (SMA), myogenin and myogenic differentiation 1 (MyoD1).

**Figure 3:**
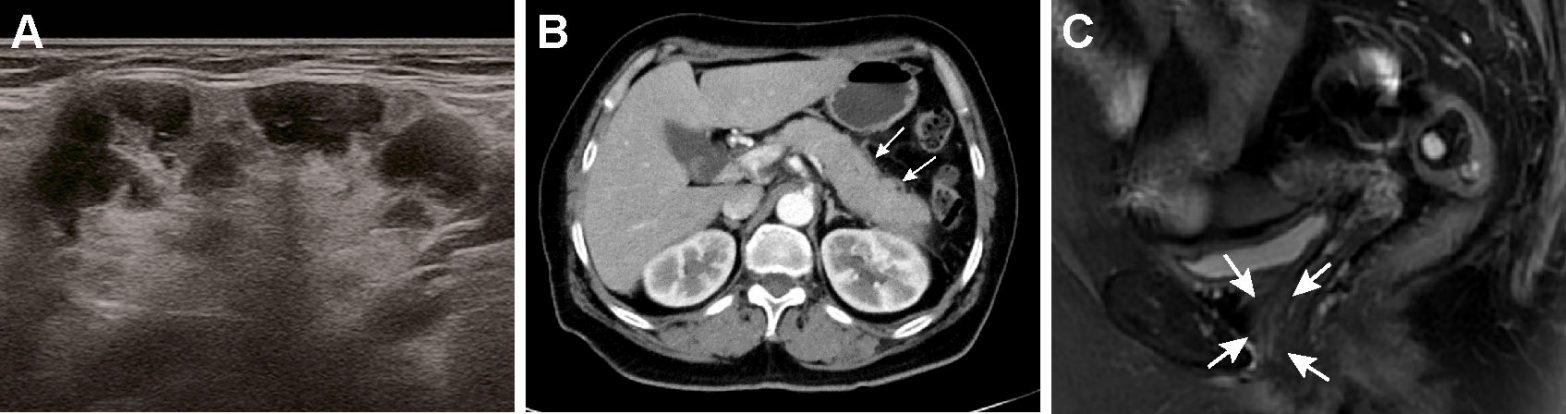
Radiological manifestations of the salivary glands and pancreas and significant improvement of periurethral mass after treatment. **(A)** An ultrasound image of the right submandibular gland showing numerous patchy hypoechoic regions inside the glandular parenchyma. **(B)** Contrast-enhanced CT scan of the abdomen showed the pancreas appears diffusely enlarged with a hypodense peripancreatic rim (white arrows). **(C)** After 3 months of treatment, MRI of the pelvis revealed marked decrease in size of the periurethral mass (white arrows).

Given the aforementioned histological findings, the patient was referred to our inpatient department with suspect of IgG4-RD. The serum IgG4 subtype test revealed a markedly elevated IgG4 level of 2640 mg/dL (normal range 3-201 mg/dL). The results of submandibular glands ultrasound showed that the echoes were uneven and there were numerous patchy hypoechoic regions inside the glandular parenchyma (**Figure 3A**). Contrast-enhanced computed tomography (CT) scan of the abdomen showed diffuse enlargement of the pancreas with delayed rim enhancement, which was in accordance to the radiologic manifestations of autoimmune pancreatitis (**Figure 3B**). A diagnosis of IgG4-RD with multiple organ involvement including lacrimal and submandibular glands, pancreas and urethra was ultimately established according to the 2019 ACR/EULAR classification criteria (5). The patient initially started 0.6 mg/kg/day of methylprednisolone (methylprednisolone 40 mg) intravenously for a week. And then she received oral methylprednisolone 40 mg once a day instead which was gradually tapered in combination with intravenous cyclophosphamide administration (0.6 g/month, 3 g total dose). Her symptom of dysuria improved greatly after steroid treatment was started and disappeared during the first week of therapy. The serum level of IgG4 was reduced to 608 mg/dL and follow-up pelvic MRI showed the periurethral lesion decreased in size substantially after 3 months of therapy (**Figure 3C**). The patient has been followed up in our clinic for 1 year and showed no signs of recurrence in the recent visit.

## Discussion

IgG4-RD is a complex multi-system disorder with distinct pathological, serological, and clinical manifestations. The clinical manifestations of IgG4-RD are widely diverse and can occur in multiple organs simultaneously or metachronically over months to years. Most patients with IgG4-RD have subacute or chronic symptoms, which depends on the organ location and usually originate from the mass effect of involved organs. It can manifest in a variety of ways, ranging from mild localized symptoms to severe tissue damage and subsequent organ failure (6). And due to the mass-forming nature of the condition, it is frequently misinterpreted as a malignant tumor, leading to unnecessary surgical therapy. The presence of elevated serum IgG4 concentration is of great diagnostic significance, but it is not necessary or sufficient for the diagnosis because up to 30-50% of patients have normal IgG4 serum levels (7, 8). Typically, IgG4-RD is confirmed and diagnosed *via* histopathology revealing significant lymphoplasmacytic infiltration, storiform fibrosis, and obliterative phlebitis as its three key pathologic features. In addition, other histological characteristics of IgG4-RD include eosinophil infiltration and phlebitis without obliteration (9). In 2019, The ACR/EULAR classification criteria for IgG4-RD were proposed to compile the disease’s clinical, serological, radiological, and pathological characteristics (5). Our patient had typical organ involvement, including lacrimal glands, salivary glands and pancreas. And the case satisfied the classification criteria with total inclusion points adding up to 43. The quick response to glucocorticoids further supported the diagnosis of IgG4-RD.

Notably, the patient had urethral involvement with dysuria as the predominant symptom, which was uncommon and easily missed. In 2012, Yamamoto H et al. firstly reported a case of urethral IgG4-RD in an elderly woman, which resembled, at first glance, an enlarged prostate in man on MRI images (10). Lately, few cases were reported. But, to our knowledge, the published literature concerning urethral IgG4-RD is rare, with only six documented cases available from PubMed so far (10–14). A summary of these cases, including ours, is presented in **Table 1**. All of the patients were female and presented at an average age of 74.4 ± 8.2 years. Except for one patient who had no symptoms, almost everyone else had urinary retention or dysuria. The majority of cases revealed multi-organ involvements, with the exception of one case that showed only urethral involvement on a positron emission tomography with computed tomography (PET-CT) scan. Within these patients, the most frequently involved organs were pancreas and salivary glands, and two of them had been diagnosed with autoimmune pancreatitis 2 years before presentation. Five cases (including ours) had IgG4 serum levels determined and all of the results were above the upper limit of normal.

**Table 1:** Published reports of IgG4-RD patients with urethral involvement.

Authors*/* publication year	N	Age/ Gender	Presentation	Extra-urethral manifestations	Imaging methods	Diagnosis	Number of IgG4, ratio of IgG4/IgG	Serum IgG4 (mg/dL)	Treatment	Follow-up
Yamamoto H et al., 2012 (10)	1	79/ Female	Urinary retention	Salivary, lacrimal glands	PET-CT, MRI, US	Biopsy	32/HPF, 41.2%	1210	GC	5 months, in remission
Choi JW et al., 2012 (11)	1	72/ Female	Dysuria	Pancreas, eyelid	MRI, CT, US	Biopsy	>30/HPF, -	NA	GC	3 months, in remission
Sangsoad P et al., 2019 (12)	1	75/ Female	Urinary retention	Pancreas	MRI, US	Biopsy	>50/HPF, 50%	NA	GC + AZA	few months, in remission
Zhang Z et al. 2020 (13)	1	75/ Female	Urinary retention	Bladder	MRI	Surgical resection	- >40%	3670	Surgical resection + GC	20 months, in remission
Murata S et al., 2021 (14)	2	67/ Female	Dysuria	None	PET-CT, MRI	Biopsy	>50/HPF, >50%	572	GC	2 years, in remission
		89/ Female	Incidental	Salivary glands, pericardium, renal pelvis	PET-CT US	Biopsy	>10/HPF, >70%	895	None	-
Our case	1	64/ Female	Dysuria	Pancreas, salivary and lacrimal glands	MRI, US, CEUS	Biopsy	>50/HPF, 55%	2640	GC + CTX	1 year in remission

N, number of patients; PET-CT, positron emission tomography with computed tomography; CT, computed tomography; MRI, magnetic resonance imaging; US, ultrasound; CEUS, contrast-enhanced ultrasound; NA, not accessible; GC, glucocorticoids; AZA, azathioprine; CTX, cyclophosphamide.

Additionally, the patient had a history of asthma and increased serum IgE levels. It should be noted that a high percentage of IgG4-RD patients report an allergic background, such as asthma, chronic sinusitis, eczema and peripheral eosinophilia (15–18). Some patients even present with severe and refractory asthma as the initial manifestation before the full IgG4-RD disease phenotype emerges or is recognized (19). The relationship between IgG4-RD and allergy is both intriguing and controversial. As IgG4-RD and allergy both engage similar Type2 immune pathways and share certain immunopathological features, it is thought that allergy plays a significant role in the pathogenesis of IgG4 (20). However, eosinophilia and elevated IgE concentrations have also been observed in IgG4-RD patients without allergic diseases (21–23), indicating that Th2 immune activation is not necessarily allergy-mediated and may be driven by immune processes inherent to IgG4-RD itself. Another study found similar IgE concentrations and eosinophil blood counts in IgG4-RD patients, regardless of history of atopy (15). Hence, further studies are required to uncover the mechanisms by which asthma is involved in the pathogenesis of IgG4-RD.

To date, data on imaging manifestations of IgG4-RD of the urethra are limited and unspecific. Typical imaging finding of urethral IgG4-RD reported in the literature was a solitary well-defined mass around the urethra while Zhang Z et al. reported a case of IgG4-RD affecting bladder wall and urethra at the same time (13). MRI was applied most frequently to evaluate the lesion and T2-weighted MRI showed different signal intensity (hypointensity or iso- to slightly hyperintensity) with variable contrast enhancement (11, 13). Three cases also used PET-CT, which revealed aberrant fluorodeoxyglucose uptake in the periurethral mass. In contrast to the poor vascularity reported by Choi JW et al. (11), we detected abundant blood flow in the mass by transvaginal ultrasound. In addition, we firstly performed CEUS to assess microvascular structure of the urethral IgG4-RD mass. CEUS is a novel tool to detect microcirculation of lesions and plays a great role in discriminating between benign and malignant tumors (24). Lesions that are enhanced homogeneously or rarely on CEUS tend to be benign. However, to our disappointment, the results of CEUS examination showed overall heterogeneously high enhancement in the lesion, which pointed towards malignancy probability and was of limited differential diagnostic value. Identifying urethral IgG4-RD from periurethral masses remains challenging and is still an unexplored field from a radiologic point of view.

It is remarkable that all previously recorded patients, with the exception of one who had surgery due to a clinical suspicion of bladder cancer, underwent ultrasound-guided transvaginal urethral biopsy, just as our patient did. Among them, five specimens revealed an IgG4/IgG ratio of more than 40% and one even higher than 70%. Thus, ultrasound-guided transvaginal urethral biopsy is highly effective and safe and should be considered as a crucial tissue sampling method for a definitive diagnosis of a periurethral mass.

In terms of treatment, except for one asymptomatic patient, all documented cases utilized glucocorticoids as the initial treatment and only one patient received azathioprine as a supplementary steroid‐sparing medication. Glucocorticoids are the first‐line agents to induce remission in patients with IgG4‐RD. Although almost all IgG4-RD patients respond excellently to glucocorticoids, disease flares are prevalent during tapering, and nearly 40% of patients fail to achieve remission after 1 year (25, 26). Therefore, prevention of IgG4-RD relapse is the focus of maintenance therapy and many immunosuppressant agents, such as cyclophosphamide, mycophenolate, azathioprine and cyclosporine have been combined with glucocorticoids to manage the disease and reduce its recurrence. Two clinical studies have proven the effectiveness of cyclophosphamide and mycophenolate in lowering the recurrence rate in 1 year to 12% and 20%, respectively (27, 28). And the B cell-depleting monoclonal antibody, rituximab has also been extensively used in IgG4-RD patients with a high rate of clinical responses. Data from uncontrolled, non-randomized prospective and retrospective studies indicate that rituximab leads to disease remission in 67-83% of the cases, allowing early tapering of glucocorticoid therapy (29–31). Because of economic considerations, the patient in our case was treated with glucocorticoids and cyclophosphamide and had no indications of recurrence after 1 year of follow-up.

## Conclusion

In conclusion, we present a rare case of IgG4-RD with multi-organ involvement including a periurethral mass which mimics malignant periurethral tumor. IgG4-RD is a systemic disorder potentially involving urethra, and is often ignored due to its rarity. It should be considered in the differential diagnosis especially when typical morphologic traits involving several organs or anatomical sites are present. The application of ultrasound-guided transvaginal biopsy and early engagement of pathologists and rheumatologists may help to diagnose the disease to avoid unnecessary surgery.

## Ethics statement

Written informed consent was obtained from the patient for the publication of any potentially identifiable images or data included in this article.

## Author contributions

ZZ participated in the management of the patient and wrote the manuscript. XL designed the study, treated the patient and revised the manuscript. SG participated in the management and follow-up of the patient. All authors contributed to the article and approved the submitted version.
